# Mitigating Ion Migration with Alternating Voltage for Stable Perovskite Image Sensors

**DOI:** 10.1021/acsami.5c18552

**Published:** 2025-12-10

**Authors:** Sergey Tsarev, Yuliia Kominko, Kyuik Cho, Lorenzo J. A. Ferraresi, Gebhard Matt, Kostiantyn Sakhatskyi, Volodymyr Svintozelskyi, Daria Proniakova, Taekwang Jang, Maksym Kovalenko, Sergii Yakunin

**Affiliations:** † Laboratory of Inorganic Chemistry, Department of Chemistry and Applied Biosciences, ETH Zürich, Zürich CH-8093, Switzerland; ‡ Laboratory for Thin Films and Photovoltaics, Empa − Swiss Federal Laboratories for Materials Science and Technology, Dübendorf CH-8600, Switzerland; § Laboratory of Integrated Systems, Department Information Technology and Electrical Engineering, 27219 ETH Zürich, Zürich CH-8092, Switzerland

**Keywords:** metal halide perovskites, ion migration, CMOS, photodetectors, stability, arrays, alternating bias

## Abstract

Perovskite photodetectors have emerged as a potential replacement for silicon photodiodes in modern cameras due to their high sensitivity to visible light and ability to be easily integrated into existing electronics. However, the use of perovskite photodetectors in conventional CMOS image sensors requires the application of reverse bias, which can lead to unstable detector performance due to ion migration effects. In this article, we propose a new approach that involves the application of forward voltage pulses to attenuate ion migration while still enabling the capture of photocurrent under reverse bias. Our results show that using this technique after each cycle of signal integration allows for stable operation of perovskite photodetectors for over 180 h, while applying a constant reverse bias leads to degradation within just 10 min. Additionally, we demonstrate stable imaging using alternating voltage and 8 × 8 crossbar arrays of perovskite photodetectors.

## Graphical abstract

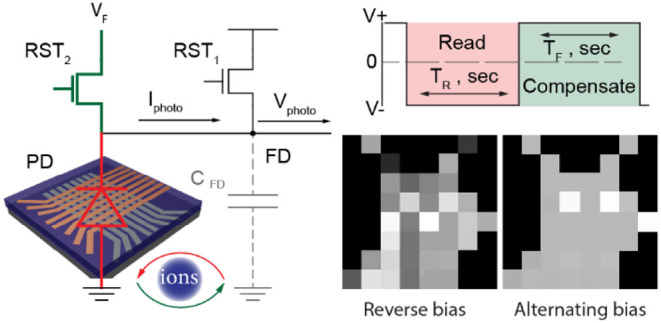

## Introduction

Image sensors are a crucial component of digital cameras, which are used widely in modern technology. The most common image sensor configuration is a 2D silicon photodiode matrix integrated into a CMOS (Complementary Metal-Oxide-Semiconductor) chip. However, silicon-based image sensors suffer from optical losses due to undesired absorption of blue light in the p+ doped region on pinned photodiodes,^1,2^ low fill factors in front-illuminated sensors,^3^ and insufficient absorption of red light in the p–n junction of the photodiode.^4^ These problems can be eliminated by separating the circuitry of the image sensor from the photoactive part and placing the photoactive, absorptive, and highly sensitive semitransparent cells on top of the silicon readout.^5,6^ Such materials should offer excellent optoelectronic properties, high responsivity, and CMOS compatible processing. In particular, lead halide perovskite (LHP)-based photodetectors demonstrate near-ideal internal quantum efficiency^7^ with response times and dark currents comparable to the state-of-the-art silicon counterparts.^8–12^ Furthermore, such detectors leverage advantages of perovskites, such as simple processing,^13–9^ band gap tunability,^17–10^ and defect tolerance.^20^ However, the ionic mobility in perovskite semiconductors, structural lability, and low thermodynamic stability^21,22,12^ raise concerns about the durability of perovskite-based optoelectronic devices.^23–25^

The instability of perovskites has already been demonstrated for solar cells and LED applications.^26,27,16,28^ LHPs are sensitive to atmospheric moisture, light, heat, and electric fields.^29^ The environmental factors can be mitigated through encapsulation.^27,30^ Thermal and photodegradation may not be as relevant for most image sensors, which are typically operated under mild conditions. The instability of perovskites under an electric field, however, can be a significant obstacle to their use in photodetectors.^31^ Recent studies have shown that mobile point defects within the perovskite lattice,^32^ such as halide vacancies and interstitials,^33^ can drift efficiently within the perovskite when the device is subjected to an electric field, leading to memory,^34^ hysteresis,^35^ and performance instability effects.^36–38^ In particular, reverse-biased perovskite photodetectors can degrade for a number of reasons, including the formation of doped perovskite interfaces with the undesired majority carrier type,^39,40^ hole tunneling due to band bending near an electron-transporting interface,^25^ and electrochemical oxidation of iodine ions.^23,41^ Modern CMOS image sensors, however, operate photosensitive elements under constant reverse-bias conditions, precluding the straightforward integration of perovskites in this technology.^4,42^

In this study, we propose a charge collection technique and the respective read-out circuit to mitigate the ion-migration-induced instability of thin-film LHPs, thereby paving the path to their deployment in image sensors. Specifically, the approach involves periodic biasing of the detectors in the forward and reverse directions while maintaining an average near-zero field across the perovskite diode. The novel read-out circuit (ROIC) design effectively stabilizes the detectors, enabling their integration into CMOS imaging electronics. Furthermore, we demonstrate the stable operation of a 64-pixel perovskite photodetector array under forward and reverse voltage pulses without sufficient degradation over 180 h, while similar reference samples operated at constant reverse bias of similar voltage degraded after first minutes of operation.

## Results and Discussion

We began with studies into various operation modes of perovskite photodetectors in order to optimize their stability, performance, and compatibility to build arrays. We thus employed a planar p–i–n diode configuration of the photosensor (Figure 1a), with PTAA (poly­(triaryl amine)) and C60/BCP (bathocuproine) as charge transport layers and an MA-free perovskite (FA_0.88_Cs_0.12_PbI_2.55_Br_0.45_) as the absorber. The latter was deposited as compact, pinhole-free, 500 nm-thick layers (Figure 1a). This configuration has been reported to exhibit relatively low dark currents^9^ and high quantum efficiency over a broad spectral range (ca. 80%), on par with our results (Figure 1b and Figures S1–S3).

**Figure 1:**
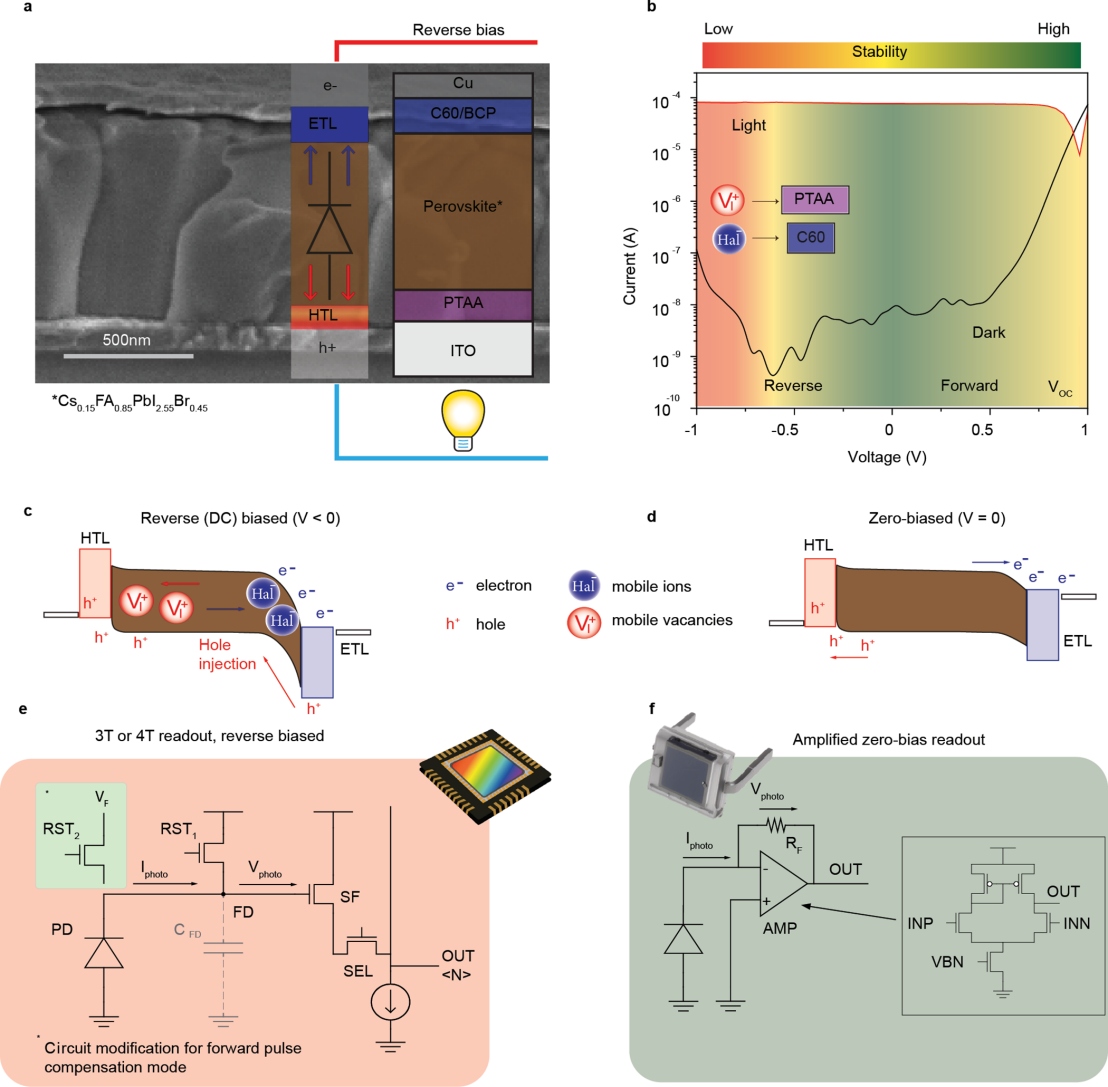
Ion migration in perovskite photodetectors. (a) Layout of a p–i–n perovskite photodiode. Scale bar is 500 nm. (b) Typical *I–V* dependencies of perovskite photodetectors in the dark (black curve) and under light (red curve). (c) Band diagram of a perovskite photodiode under applied reverse bias and (d) in photovoltaic mode (zero bias). e^–^,h^+^: charge carriers, colored circles: mobile ions. (e) 3T (or 4T with an additional transistor) readout electronic circuit for reverse-bias operation of the photodetector. Reset voltage (RST) is used to charge the photodiode (PD), following photocurrent (Iphoto) discharging floating diffusion capacitance (FD). The charge resulting from this charge is used to bias the source follower (SF) transistor, following by the pixel selection (SEL) transistor. An additional transistor highlighted with a green background supplies a compensational voltage pulse, as discussed further below. (f) Electronic readout circuit for zero-bias operation of the photodetector.

The current–voltage characteristics of the photodetector measured under dark and illuminated conditions (1 mW/cm^2^, red LED) are shown in Figure 1b. Perovskite photodiodes typically exhibit poor stability under reverse bias due to ion migration toward charge transport interfaces, as illustrated in Figure 1b,c. Assuming that halides are the dominant type of mobile ions in the perovskite films, the halide vacancies, under the reverse bias, move toward the anode (ITO electrode), and halide anions migrate toward the cathode (copper). The resulting accumulation of ions near the electron-transporting interface leads to intense band bending and hence a drastic increase of the dark current due to the holes tunneling through the electron transport layer, as discussed in the literature.^25,33,43^

Methylammonium (MA)-based perovskites are reported to be the most susceptible to ion migration, while substituting MA content with cations with smaller dipole moments results in more stable compositions.^44^ To confirm this, we fabricated and tested the extent of polarizability in photodiodes with conventional MAPbI_3_^25^ and^39^ MA-free Cs_0.15_FA_0.85_PbI_2.55_Br_0.45_ active layers. The device with MAPbI_3_ sandwiched between PEDOT: PSS and Au had completely switched the diode direction when polarized at −2 or 2 V for 1 min (Figure S4). Despite the lower photocurrent magnitude and fill factor, the device with a mixed cation composition also showed permanent polarization and switching behavior.

Notably, when the device is reverse-biased relative to a present state, it switches to an opposite diode direction, effectively becoming forward-biased once switched. This was interpreted as the creation of doped perovskite interfaces near the electrodes, leading to an n-doped perovskite interface near the hole-collecting electrode and *vice versa*, which is undesirable for proper photodiode function.^39^ The ion migration is also evident from the frequency-dependent capacitance spectra, matching the other reports.^45^ Furthermore, the devices exhibited a permanent decrease in photoluminescence after applying reverse bias for 5 min at −3 V.

On the contrary, field-related ion migration is minimal, and the photocurrent is most stable in the photovoltaic mode (Figure 1d, also discussed further). Unsurprising is seeing perovskite photodetectors commonly operated at 0 V bias.^8,46–49^ At the same time, the integration of perovskite active layers with ROIC chips has not been reported. Standard ROIC configuration requires operation at sufficient values of reverse potential (Figure 1e), which alone can preclude the deployment of perovskites in image sensors. While 0 V operation (photovoltaic mode) is well-suited for single-channel operation, its implementation in compact high-resolution image sensors is a formidable challenge. In the photovoltaic mode, the current signal magnitude falls within the picoampere range owing to the microscale active area of pixels. Such small signal amplitude would require integration of transimpedance amplifiers into each pixel (Figure 1f). This scheme requires at least 5 additional transistors, occupying a relatively large area for each pixel and consequently limiting the attainable image sensor resolution as well as sensitivity. Each of the transimpedance amplifier circuits uses a resistor, whose fabrication introduces a large pixel-to-pixel variation in device parameters. Transimpedance amplifiers consume a significant amount of power, which will then scale with the number of pixels per unit area.

One of the most commonly used basic ROIC circuits for image sensors is called a 3T circuit (i.e., includes 3 transistors per pixel to integrate the charges generated by the photodiode and drive them). In this circuit, the detector is constantly under reverse voltage during the acquisition period (Figure 1e, area highlighted with orange). The 3T or similar readout circuits are utilized in most image sensors due to low noise, relative ease of fabrication, and low power consumption. In this case, the charge is converted into an easy-to-read millivolt range voltage amplitude. The primary hurdle to overcome is thus to ensure the stability of the perovskite layer under reverse bias conditions in a standard 3T ROIC configuration. We reasoned that the alternating voltage pulses can prevent ion accumulation at the electrodes owing to the limited ion-drift in both directions.^50^ A forward voltage pulse (compensation pulse) was thus applied for the recovery of the ionic state of the device, each time after an acquisition period under the reverse bias (reading pulse), as shown in Figure 2a,b.

**Figure 2:**
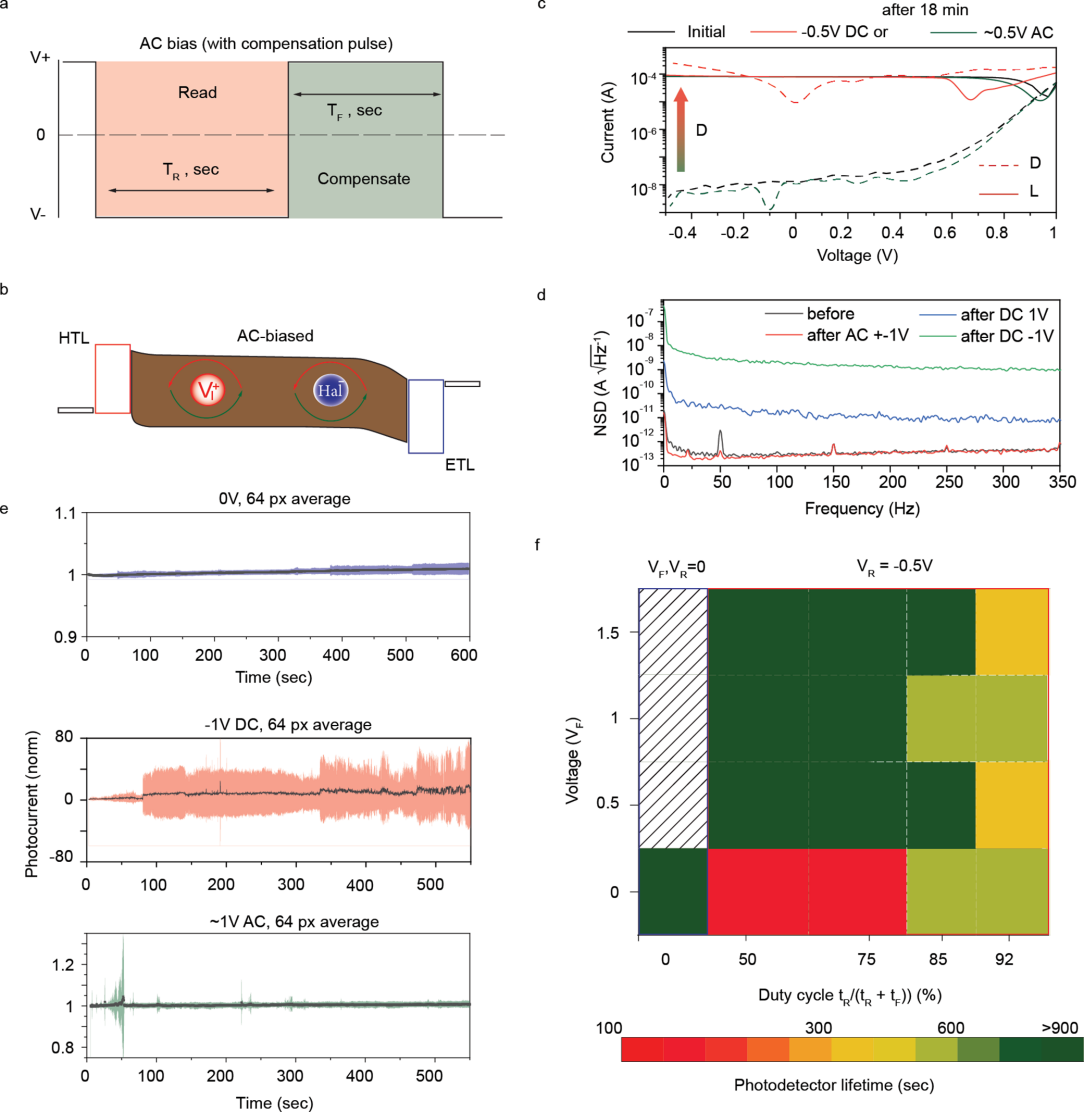
Characterization of perovskite photodetector stability under zero, alternating, and reverse bias. (a, b) Voltage traces for AC bias operation and a band diagram of the perovskite photodetector under alternating voltage. (c) *I–V* curves of perovskite photodetectors before and after applying reverse and alternating biases under 1 mW·cm^–2^ light. (d) Noise spectral density of detectors after biasing with 1 V reverse, forward, and alternating bias for 10 min (e) Average (64 pixels) photocurrents (normalized to the initial value) under −1 V DC, 0 V, and 1 V AC bias for 10 min. Standard deviation is depicted as colored bands around the main trace. (f) 2D map of photodetector lifetimes vs duty cycle of the reverse pulse (*x*-axis) and forward pulse voltage (*y*-axis).

*I–V* characteristics in dark (D) and light (L) after 18 min of polling under reverse DC (direct current) bias evidence rapid device degradation, seen as a drastic increase in the dark current, unlike operation under AC (alternating current) voltage of similar amplitude (Figure 2c). Furthermore, alternating voltage for 10 min was found to effectively restore the performance of devices previously damaged by reverse bias (Figure S5). In addition, an application of DC-bias to devices further accelerates the degradation, unlike to AC-operated devices, illustrated with the respective increase in the noise current^51,34^ (Figure 2d). Specifically, the initial detectivity of 6.6 × 10^11^ Jones slightly increased to 9.3 × 10^11^ Jones after polarization with 1 V AC for 10 min, while it substantially degraded when poled at −1 V (to 5.6 × 10^11^ Jones) and 1 V (to 4.3 × 10^11^ Jones). These observations were statistically confirmed by analyzing photocurrent traces for groups of 64 pixels kept at 0 V, DC, or AC bias under illumination. Figure 2e shows the photocurrent (solid line) normalized by its initial value for every pixel and subsequently averaged for each measurement frame across the entire 64-pixel array, with its standard deviation being highlighted by colored bands. As expected, devices exposed to reverse bias showed significant degradation compared to the rest of the pixels. To provide quantitative analysis of AC bias pulse parameters (forward voltage and duty cycle of the reverse pulse at 20 Hz), we considered the photodetector lifetime (a period after the standard deviation exceeds 20%) as a figure of merit. The stable operation range was determined to be up to 80% of the duty cycle, independent of the amplitude of the forward voltage, as shown in Figure 2f.

To gain deeper insights into the changes in mixed cation perovskites under AC and DC bias, we deposited perovskites on substrates with lateral interdigitated electrodes and polarized them using either 20 V DC or ±20 V alternating bias (Figure 3a). The application of DC voltage was expected to induce halide migration toward the positive electrode, resulting in the formation of PbI_2_/PbBr_2_ on the negative electrode. Optical microphotographs (Figure 3b,c) support this hypothesis, showing the formation of orange-colored material on the negatively charged electrodes, consistent with reported studies.^33,39,45^ However, no visible changes were observed in the AC-poled perovskite. To confirm the self-doping effect, we measured changes in the perovskite work function across the polarized channel by using Kelvin Probe Force Microscopy. The pristine (Figure 3d) and AC-poled (Figure 3f) samples showed uniform surface potential with local minima in the middle of the channel. In contrast, the DC-poled (Figure 3e) sample displayed a nonuniform potential distribution, with a 200 mV higher surface potential near the negatively charged electrode, indicating the formation of lead halide salts known to be n-type semiconductors.^25^ The absence of lateral polarization in the AC-poled sample suggests effective suppression of ion migration.

**Figure 3:**
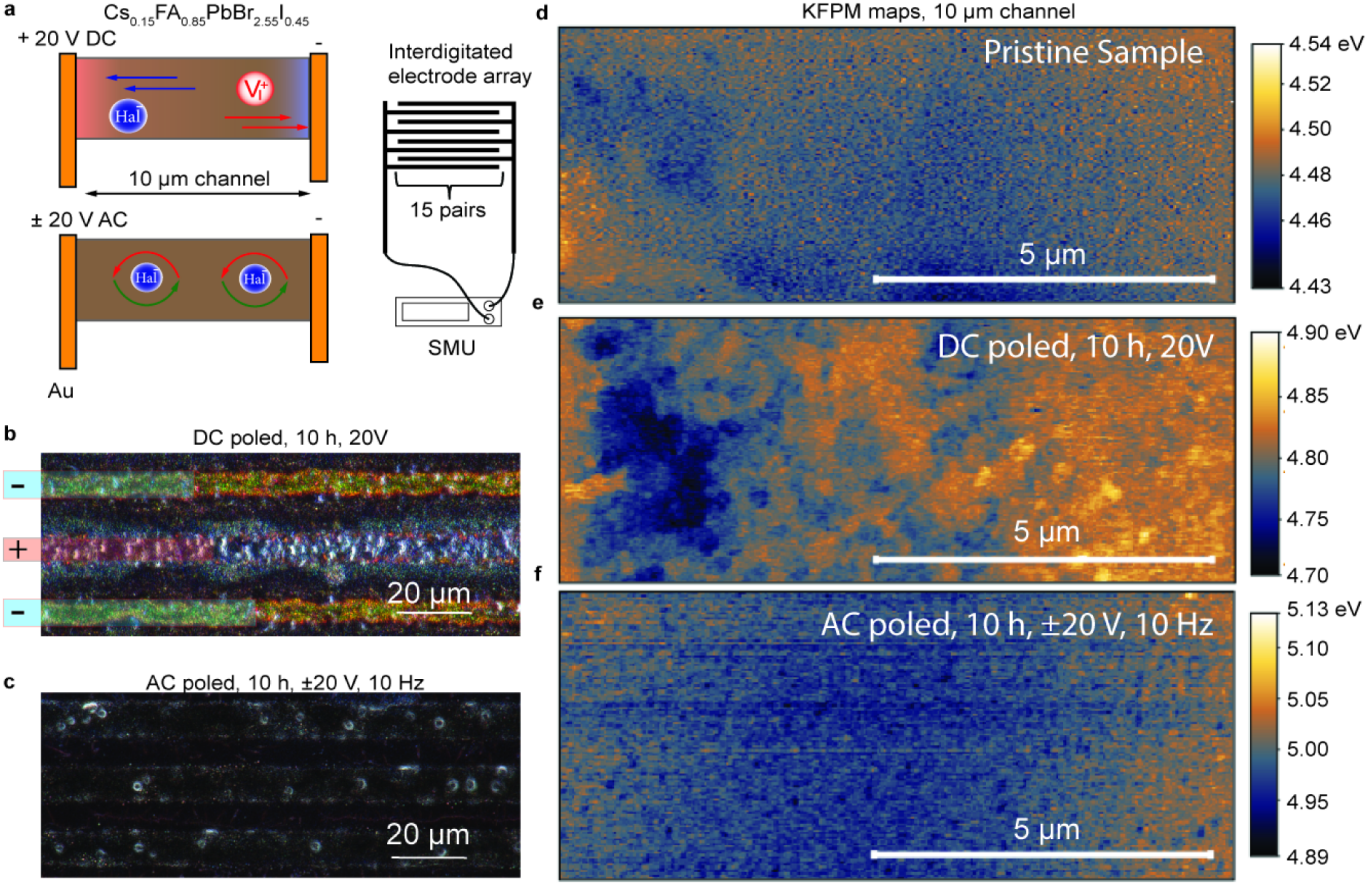
Polarization of perovskite in lateral photoconductors using interdigitated electrode arrays. (a) Measurement and sample preparation schematics. (b, c) Optical microphotographs of interdigitated electrodes after poling for 10 h under 20 V DC (b) or 20 V AC (c). The polarity of the electrodes is highlighted with blue and red semitransparent boxes. (d, e, f) Kelvin-probe Atomic Force Microscopy maps of the channels before (d) and after DC (e) and AC (f) poling.

Following the identification of the optimal parameters, we proceed with benchmarking of a related ROIC concept based on the standard 3T configuration (Figure 1e, area highlighted with orange). For this purpose, we developed a custom-built single-channel readout board (Figure S6) specifically designed for the application of voltage pulses to including an optional compensational pulse (Figure 1e, part highlighted with green). In the standard operation (SO) mode, a time diagram of the potential on the photodiode node (Figure 4a) consists of integration and reset phases. The magnitude of voltage detected at the end of each integration phase is proportional to the accumulated charge. To prepare the detector to the next acquisition frame, the detector is reset to the initial state using a reverse bias pulse. During the entire frame (integration + reset), the perovskite photodetector is under the reverse bias, causing instability issues discussed above. To mitigate the ion-migration-related effects, we introduced an additional phase of a compensational pulse (i.e., forward pulse operation (FPO) mode, illustrated in Figure 4b) after an integration phase. During the FPO operation, the integration time was set up as 50% from the total frame time, while the combined duration of compensation and reset time accounted for the remaining 50% of the frame. In contrast, in the SO mode, the integration time comprised 85% of the total frame time.

**Figure 4:**
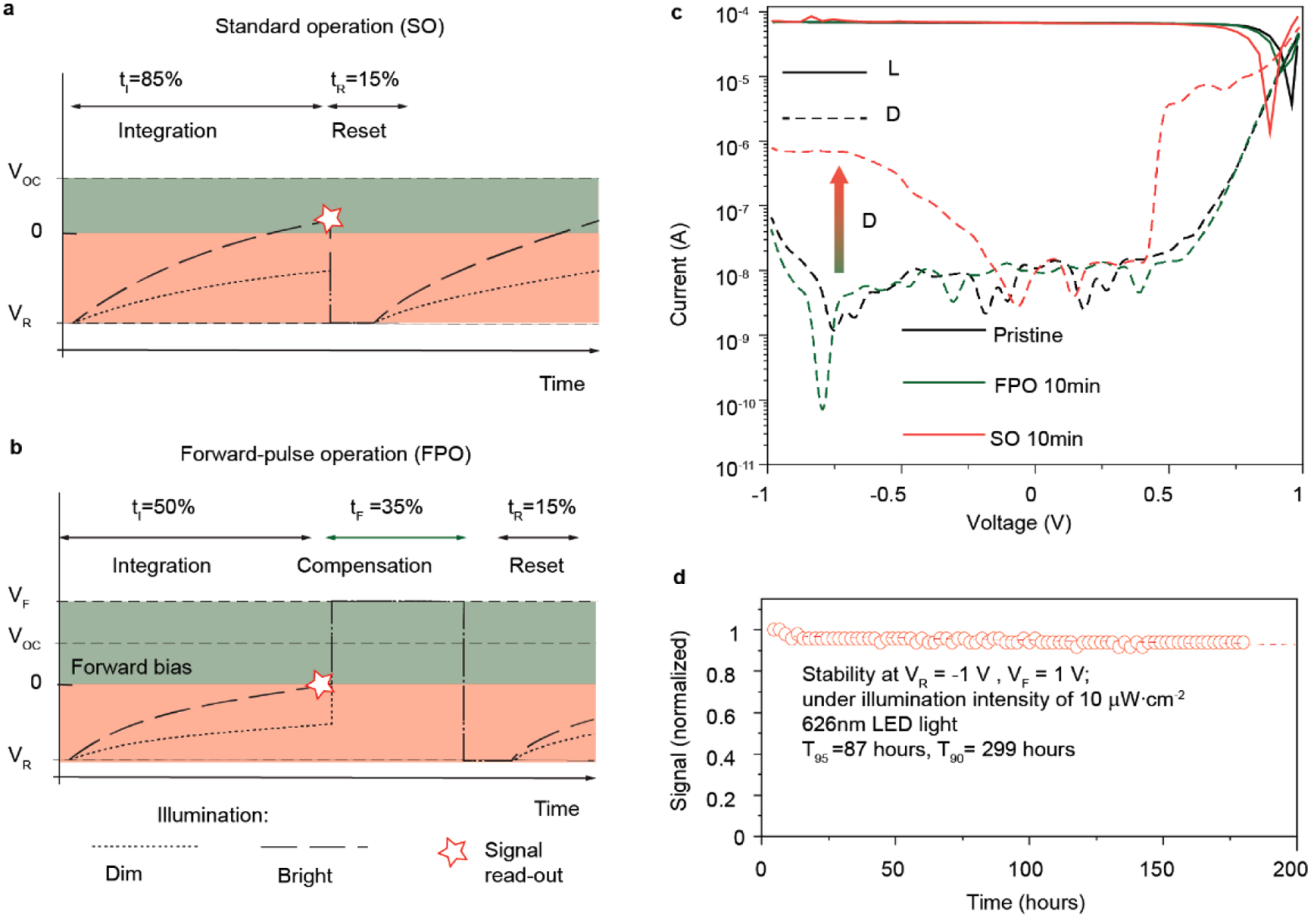
Perovskite photodetector operation using a single-channel readout board. (a, b) Time diagrams of potential on the photodiode node during standard operation (SO, a) and forward pulse operation (FPO, b). (c) *I–V* characteristics of the perovskite photodetector before and after application of FPO and SO for 10 min. (d) Long-term stability of the device during FPO.

Figure 4c shows *I–V* characteristics indicating an increase of the dark current for perovskite photodetectors after 10 min of degradation using the SO protocol, while no change in device characteristics was observed if the FPO protocol was used (more systematic results with variations in bias voltages are presented in Figure S7). Although the degradation under SO was not as dramatic as during DC operation, it is likely due to the lower averaged value of bias during the integration cycle compared to the voltage applied during the DC bias poling (Figure 2c,e). While being operated in the SO mode, pixels tend to degrade within the first tens of minutes, whereas the FPO showed much higher stability with a *T*_90_ of 299 h extrapolated from a linear fit during the 180 h of constant operation test, measured as voltage read-out from the single-channel board (Figure 4d). We observed only a slight decrease in signal magnitude, which was attributed to light-induced effects or interfacial degradation over time.

Inspired by the results above, we demonstrated the implementation of the FPO readout circuit in an 8 × 8 crossbar perovskite photodetector array (Figure 5a). To test the array, a customized switching board that included fast switches controlled with an Arduino board was used (shown in Figure 5b). The simplified circuit diagram of the board is shown in Figure 5c. Photoresponse traces from each pixel during SO and FPO were combined in a video file to visualize the perovskite photodetector degradation processes (Videos S1 and S2). Snapshots collected after 0, 10, 60, and 600 s from the start of testing are highlighted in Figure 5d,f. Figure 5e,g shows photoresponse (solid line) normalized by its initial value for every pixel and subsequently averaged for each measurement frame across the entire 64-pixel array, with its standard deviation being highlighted by colored fields. The statistical representation of degradation effects emphasizes the significant difference in signal stability observed for perovskite photodetectors when operated in SO or FPO modes.

**Figure 5:**
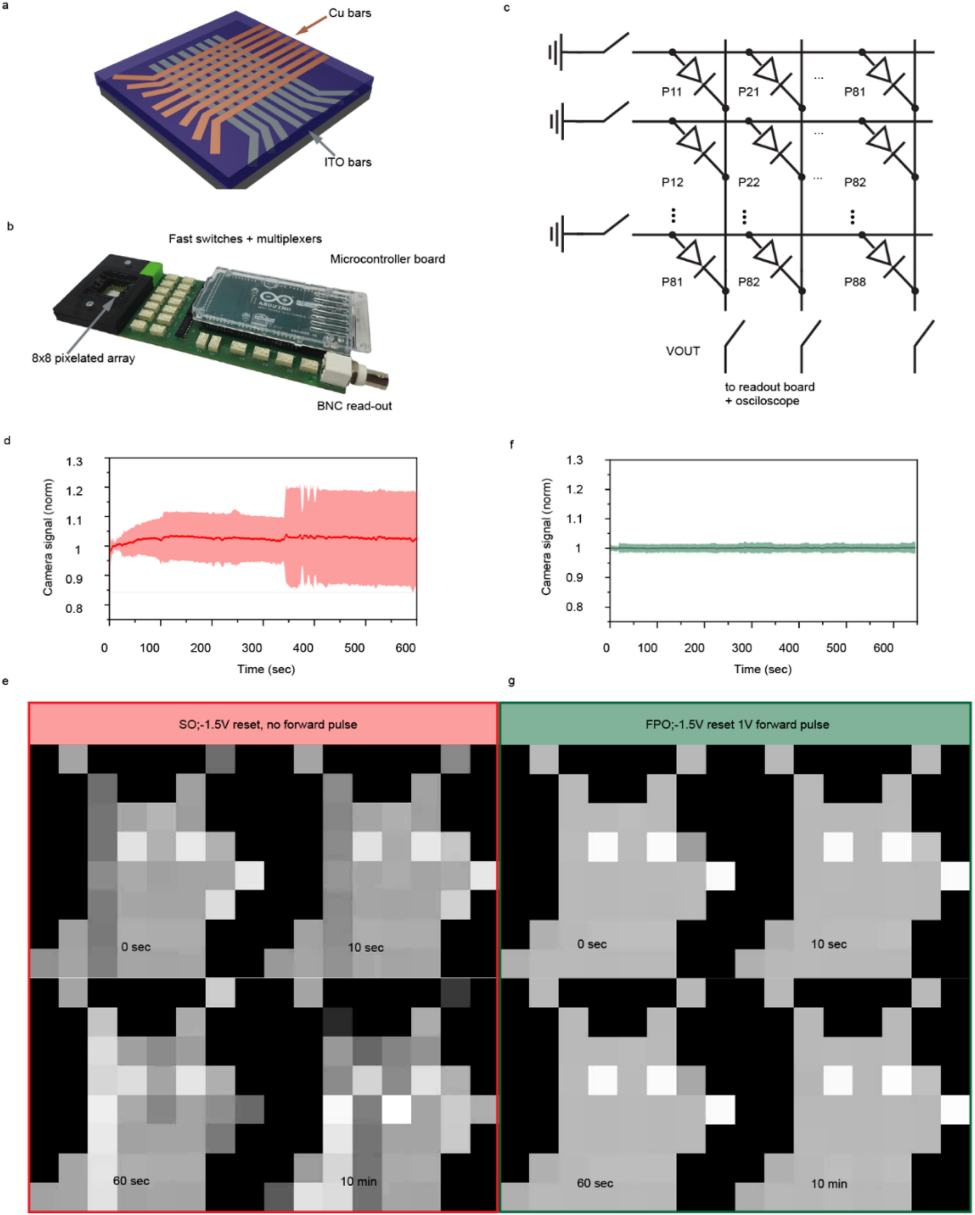
Stability and imaging with perovskite photodetector arrays. (a) Schematics of a perovskite 8 × 8 crossbar photodetector array. (b) Read-out switch board for imaging. (c) Circuit diagram of the crossbar arrays. (d) Averaged normalized signal vs time from the array during SO operation. (e) Photogenerated signal data obtained from a photodetector array during SO operation, depicted in a form of an 8 × 8 pixel image. (f) Averaged normalized signal vs time from the array during FPO operation. Standard deviation is depicted as colored bands around the main traces. (g) Photogenerated signal data obtained from a photodetector array during FPO operation, depicted in a form of an 8 × 8 pixel image. The images in (e, g) are frames from Videos S1 and S2.

## Conclusion

The integration of perovskite materials into modern readout circuits is challenging due to their susceptibility to ion migration, emerging from the ionic nature of their crystalline lattice. An application of a consistent electrical field required for image sensor operation causes ion accumulation near electrodes, eventually leading to a breakdown of the diode. We demonstrate that the applied alternating bias can effectively stabilize detector performance by preventing ion movements within the device. Specifically, the detectors revealed enhanced stability under an alternating 1 V voltage, in contrast to rapid degradation within 10 min observed under −1 V constant potential.

Based on that approach, we developed a prototype perovskite photodetector array, incorporating a custom-made readout circuit that employs a compensational pulse regime. This regime applies a forward pulse following each integration period, effectively inhibiting ion migration. Our prototype demonstrated stable operation under an alternating 1.5 V bias for 180 h, showcasing the potential of this approach for high-performance image sensors.

The perovskite materials represent a new class of semiconductors with a soft ionic lattice. Consequently, their ionic conductivity often emerges as the main constraint in applications requiring stable and controllable optoelectronic characteristics. Fundamentally, our research establishes an application-focused framework that limits the influence of mobile ions and supplies practical stabilization guidelines based on electronic bias that could help improve other perovskite-based technologies like memristors and LEDs. The techniques we have developed may thus have far-reaching implications beyond the field of photodetection. Furthermore, our work marks a significant step forward in the use of perovskite materials in modern integrated optoelectronic devices. By overcoming the challenge of ion migration, we paved the way for the successful integration of perovskite photodetectors in modern image sensors, delivering superior detector performance without compromising detector stability.

## Experimental Methods

### Precursor Solution Preparation

0.6 g FAI (homemade), 1.6 g PbI_2_ (Fisher Scientific, 99.9%), 0.184 g PbBr_2_ (Acros Organics, 98%, extra pure), and 0.104 g CsBr (99.99%, Alpha Aesar) were added into a vial and dissolved in a mixture of 280 μL of DMSO (Extra-Dry, Acros Organics) and 1930 μL of DMF (Extra-Dry, Acros Organics). The prepared solution was kept on a hot plate at 80 °C until it was dissolved (normally for about 4–5 h). After the dissolution, the solution was filtered through a 0.25 μm PTFE filter and used within 20 min.

### Device Fabrication

Glass substrates coated with ITO (Zhuhai Kaivo, 18 Ω·sq^–1^) were sequentially cleaned with soap, water, acetone, and isopropanol. For array fabrication, substrates were additionally patterned using standard wet photolithography methods. After 10 min of UV/O_3_ cleaning (UV-Ozone cleaner, Ossila), the substrates were transferred into a nitrogen-filled glovebox, where 4 mg·ml^–1^ of PTAA (Lumtec) was spin-coated at 4000 rpm for 40 s and annealed at 100 °C for 10 min. The perovskite coating process started with 65 μL of DMF being dispensed on a rotating substrate at 6000 rpm. After 15 s, 65 μL of precursor solution was dropped on a rotating substrate following 150 μL of toluene dropped after 10 s. Substrate rotation stopped after 10 s following the toluene drop. The perovskite was annealed for 10 min at 100 °C. Electron transport layers were formed via the evaporation of 20 nm of C60 (Ossila) and 8 nm of BCP (Alfa-Aesar). Copper electrodes (100 nm) were evaporated through shadow masks, yielding an area of 0.16 cm^2^ for single pixels or 1 mm^2^ for 8 × 8 arrays.

### Polarization of Device with Interdigitated Electrodes

Glass substrates with Ti–Au interdigitated electrode arrays were purchased from Micrux Technologies. The substrates were sequentially cleaned with soap, water, acetone, and isopropanol. The perovskite was coated in the same manner as that used for the photodetector devices. Polarization was performed by poling the device at 20 or ±20 V with a 5 Hz frequency (100 ms duration per half cycle). Both polarizations were conducted for 10 h.

Atomic force microscopy was performed by using a Park NX10 microscope and NSC36C tips. The surface work function was calibrated by using a freshly cleaved HOPG sample. Samples were scanned at 0.2 Hz with a 1 V drive voltage in the tapping mode. Optical images were collected using a Keyence VHX Digital Microscope.

### SEM-Cross Section Measurements

SEM cross sections and images were acquired with a Zeiss ULTRA plus Digital Field Emission Scanning Electron Microscope with acceleration 1–5 kV using a secondary electron detector.

### Current–Voltage Measurements

The *I–V* curves of devices were collected with a Keysight B2920 SMU using a homemade photodetector testing setup with a red LED as a light source (emission spectrum is shown in Figure S8). The *I–V* sweeps were performed from −1 to 1 V at 200 mV·sec^–1^ rates. All *I–V* measurements were taken in a nitrogen-filled glovebox. To measure stability, the photodetectors were subjected to DC or AC bias square wave pulses with certain duty cycle parameters supplied with the SMU, while the current response was monitored. The AC bias cycle duration was fixed at 120 ms. The shape and continuity of the waveform were confirmed with a digital oscilloscope.

### Transient Photocurrent Measurements

Transient photocurrent (TPC) measurements were performed using a Becker & Hickl GmbH BDL-SMN Series 473 nm pulsed diode laser with a repetition rate of 100 kHz and a pulse width of 90 ps. The signal was amplified with a Femto HSA-X-I-2-40 wideband voltage amplifier with a 160 ps rise-fall time and 40 dB fixed gain before sending it to a Tektronix MSO44 mixed-signal oscilloscope with 500 MHz bandwidth, 160 ps resolution, and 6.25 GS s^–1^ sampling rate.

### Noise Measurements

To determine the Noise Equivalent Power (NEP) of a photodetector, the output noise spectral density $S_{n}\left(f\right)$ in $\frac{A}{\sqrt{Hz}}$ was measured with the network analyzer (SR770 FFT Stanford Research Systems), followed by a Fast Fourier Transformation (FFT). The noise spectral density was measured at −0.1 V reverse bias before and after 10 min of accelerated stability testing. As the investigated photodetectors have a low level of output noise spectral density, the variable gain low noise current amplifier was used (DLPCA-200). The NEP was determined as the output noise spectral density divided by the responsivity of the photodetector:

1 $$NEP=\frac{S_{n}}{R}\left[\frac{W}{\sqrt{Hz}}\right]$$

The specific detectivity D* is normalized to the active area representation of the NEP. It can be determined as

2 $$D^{*}=\frac{\sqrt{A}}{NEP}\left[\frac{cm\sqrt{Hz}}{W}\right]$$

here *A* is the active photodetector area in cm^2^.

### Testing Devices with a Read-Out Board

The read-out board displayed in Figure S6 was used. The voltage pulses were supplied with a function generator and monitored with a Hantek DSO5102P oscilloscope. The reset schedule was configured to supply the detector at the beginning of each frame with either solely negative short (*V*_R_ = −1.5 V, *t*_R_ = 15% of the acquisition cycle) reset pulses for SO or a sequence of negative (*V*_R_ = −1.5 V, *t*_R_ = 15%) and positive pulses (*V*_F_ = 1 V and *t*_R_ = 35%) for FPO. The frame rate was 1 kHz for both SO and FPO. The bias on the sample was monitored with a digital oscilloscope. The photodetectors were connected to the board through a shielded cable and measured in a nitrogen-filled glovebox. For array measurements, the devices were connected to the read-out board via the array switching board (Figure 5b). The photodetector arrays were exposed to a uniform light intensity of approximately 0.1 mW·cm^–2^ from a green LED, and the response of each pixel was collected for 10 min sequentially. The voltage signal from each pixel was normalized by the maximum value of the voltage trace obtained for each pixel within these 10 min. To obtain a visual representation of degradation effects (Figure 5e,g and Videos S1 and S2), the signal of each pixel was weighted to the intensity of the pictogram of a dog. For the long-term stability testing, the device was connected to the read-out board in a similar manner and the voltage signal was continuously monitored for 180 h under 0.1 mW·cm^2^ illumination at the FPO mode.

## Supplementary Material







## Notes

Imaging using SO (Video S1) (MP4)
Imaging using FPO (Video S2) (MP4)
External quantum efficiency, steady-state dark current, temporal response of the photodetectors, light source emission spectrum, IV curves of switchable photoconductor devices, capacitance vs frequency spectrum, steady-state photoluminescence after and during the application of bias, IV curves of detectors after degradation under reverse bias, and after recovery using AC bias, a photo of the electronic read-out, IV curves of devices after the application of FPO and SO (PDF)
