# Supplementary material for: Mitigating Ion Migration with Alternating Voltage for Stable Perovskite Image Sensors
Source: ACS Appl Mater Interfaces. 2025 Dec 10;17(51):69635–44. doi: 10.1021/acsami.5c18552 (PMC12754746; doi:10.1021/acsami.5c18552)
Supplement: Supplementary file 3 [file am5c18552_si_003.pdf]

Supporting information

Mitigating ion migration with alternating voltage for stable perovskite image sensors

Sergey Tsarev[1,2], Yuliia Kominko[1,2], Kyuik Cho[3], Lorenzo J.A. Ferraresi[1,2], Gebhard Matt[1,2], Kostiantyn Sakhatskyi[1,2], Volodymyr Svintozelskyi[1], Daria Proniakova[1,2], Taekwang Jang[3], Maksym Kovalenko[1,2], Sergii Yakunin[1,2*]*

[1] Laboratory of Inorganic Chemistry, Department of Chemistry and Applied Biosciences, ETH Zürich, CH-8093 Zürich, Switzerland

2 Laboratory for Thin Films and Photovoltaics, Empa – Swiss Federal Laboratories for Materials Science and Technology, CH-8600 Dübendorf, Switzerland

3 Laboratory of Integrated Systems, Department Information Technology and Electrical Engineering, ETH Zürich, CH-8092 Zürich, Switzerland

AUTHOR INFORMATION

Corresponding Author:

E-mail: yakunins@ethz.ch; mvkovalenko@ethz.ch

Table of contents

Figure S1.

Representative EQE spectrum of the photodetectors used in this study.

Figure S2.

Steady-state dark current measurement.

Figure S3.

Temporal response of the photodetectors depending on the pixel size.

Figure S4.

Ion migration assisted polarization of perovskites.

Figure S5.

Degradation and recovery of a perovskite detector under reverse bias.

Figure S6.

Photograph of the read-out board with labeled parts.

Figure S7.

I-V curves of devices after SO (a) and FPO (b).

Figure S8.

Emission spectrum of red LED used as a light source for this study.

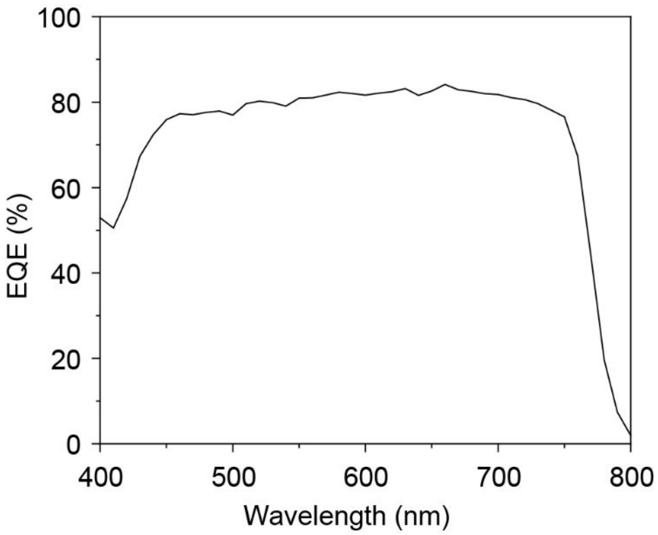

Figure S1. Representative EQE spectrum of the photodetectors used in this study The structure of the photodetectors is ITO/PTAA/$FA_{0.88}Cs_{0.12}PbI_{2.55}Br_{0.45}$/C60/BCP/Cu.

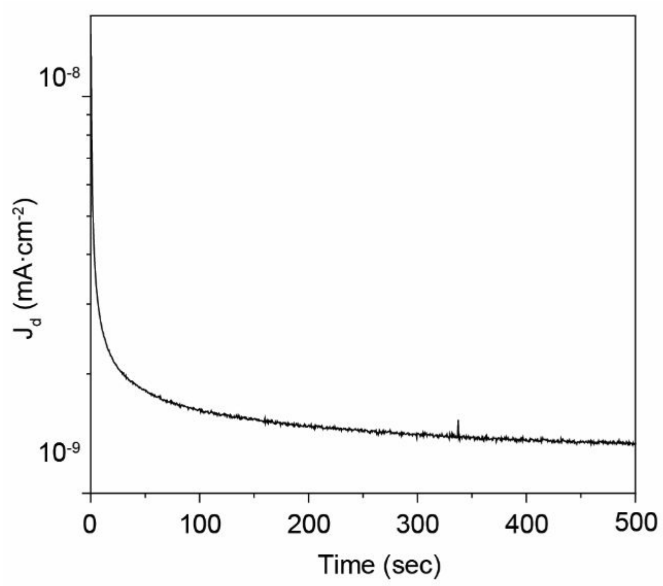

Figure S2. Steady-state dark current measurement Steady-state dark current of 2 nA·cm^{-2} was measured after 10 minutes of polling at -0.5 V in dark.

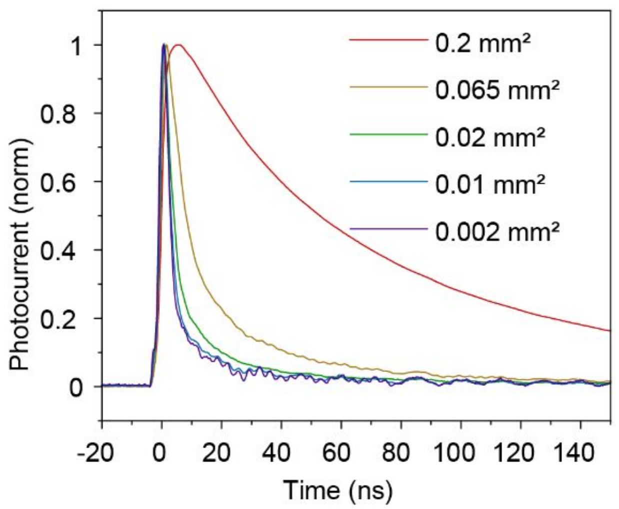

Figure S3. Temporal response of the photodetectors depending on the pixel size

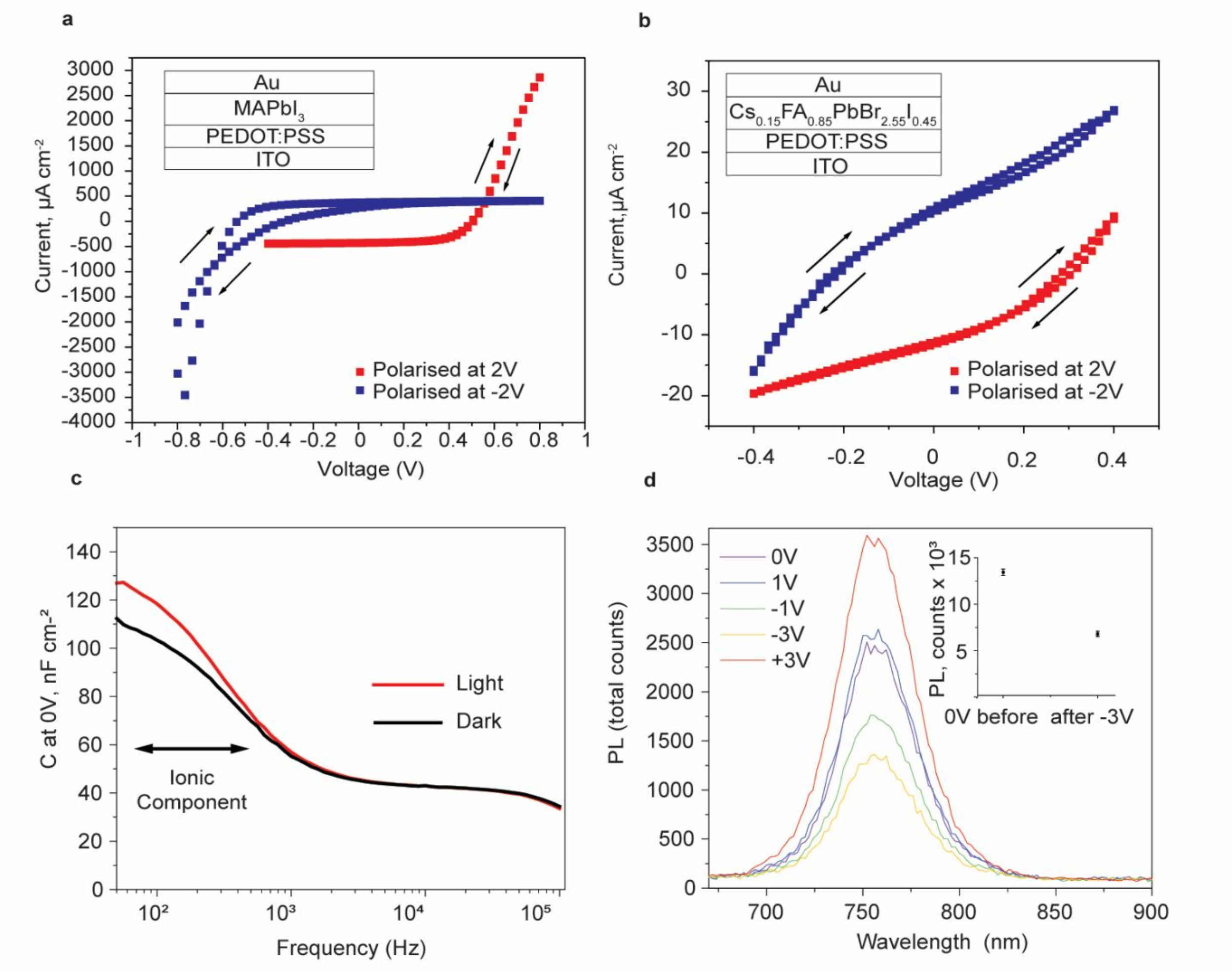

Figure S4. Ion migration assisted polarization of perovskites. a, b current-voltage characteristics of an ETL-free device with ITO/PEDOT:PSS/Perovskite/Au structure where perovskite is $Cs_{0.15}FA_{0.85}PbBr_{2.55}I_{0.45}$ (a) and $MAPbI_3$ (b) **c**. Frequency-dependent capacitance spectrum of the perovskite photodetector, measured under dark and illuminated (white LED, ~200 lm) conditions **d**. steady-state photoluminescence spectra recorded during the polarization of the perovskite photodetector under 0V, 1V,-1V, -3V, +3V for 1 min. Inset: changes in PL intensity measured at 0V before and after applying -3V for 5 minutes

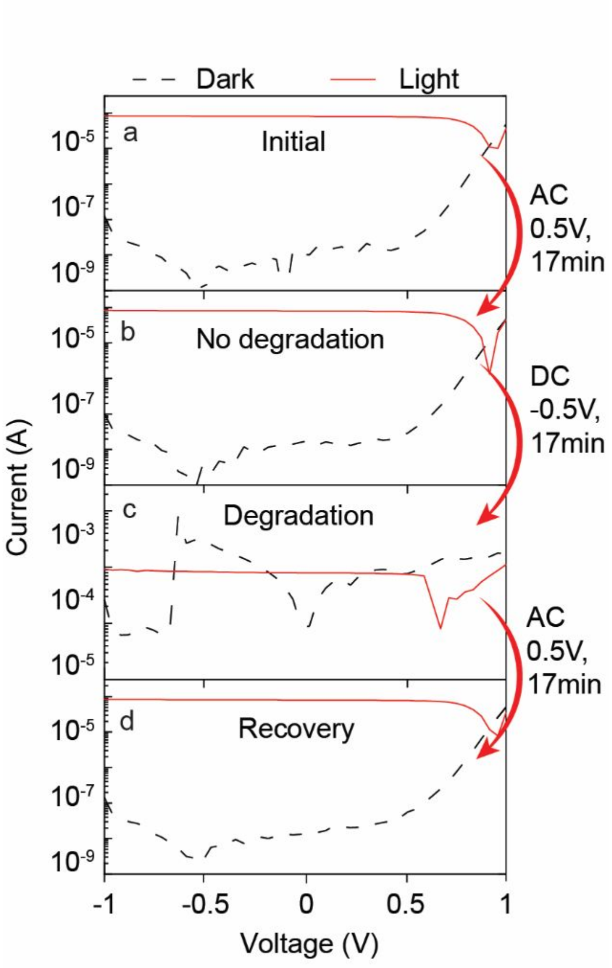

Figure S5. Degradation and recovery of a perovskite detector under reverse bias *I-V* characteristics of a perovskite photodetector in an initial state (a) and during a sequential application of AC (b), DC (c) and AC (d) bias again for 18 minutes each at -0.5 V for DC or ±0.5 V, 5Hz AC. Both degradation and recovery took place under 1mW/cm^2 626 nm red LED light

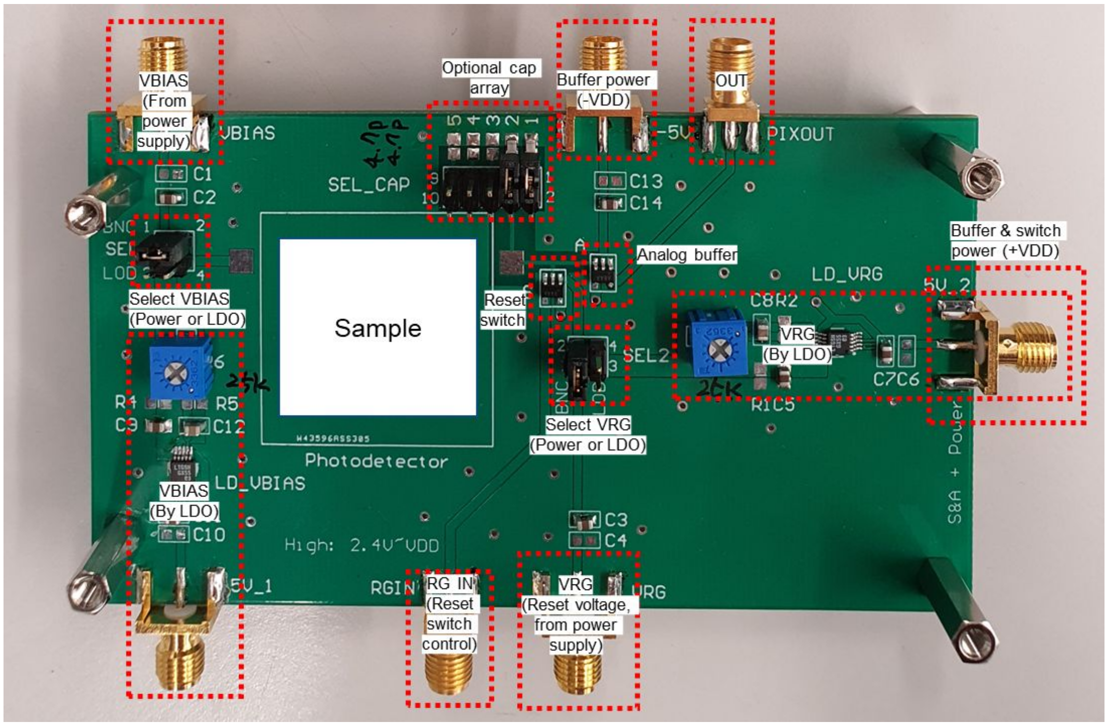

Figure S6. Photograph of the read-out board with labeled parts.

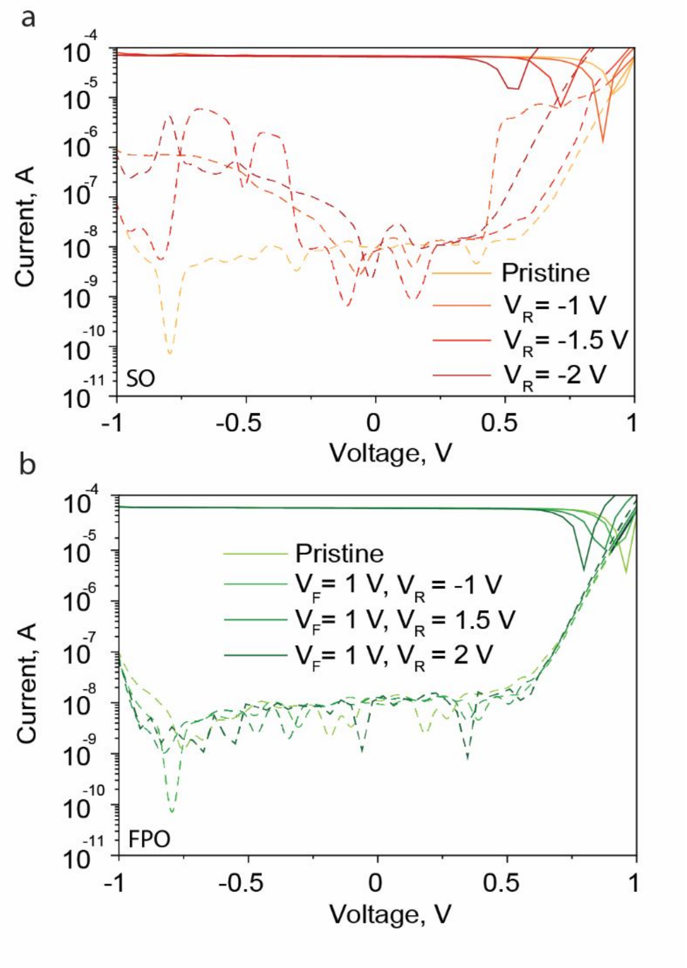

Figure S7. *I-V* curves of devices after SO (a) and FPO (b). The device was tested sequentially according to this procedure: FPO (V_F =1 V, V_R = -1 V); SO (-1V); FPO (V_F =1 V, V_R = -1.5 V); SO (-1.5 V); FPO (V_F =1 V, V_R = -2 V); SO (-2 V). A decrease in V_{OC} and an increase of leakage (dark) current was observed during the SO. The dark currents and partially V_{OC} were recovered to the previous state during the FPO operation.

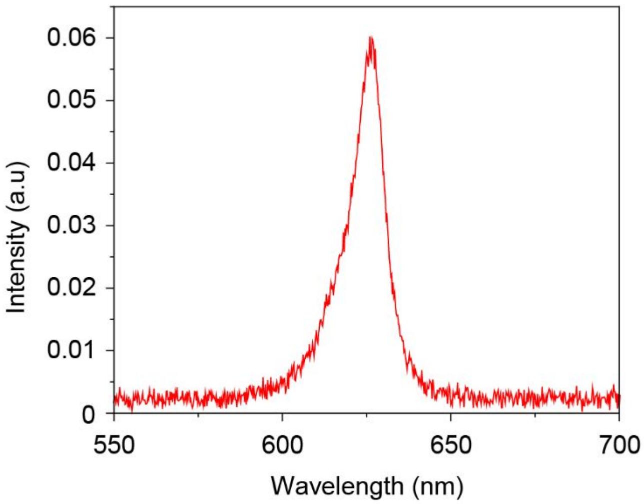

Figure S8. Emission spectrum of red LED used as a light source for this study

Supplementary Video 1. Evolution of the image captured by perovskite photodetector array in SO mode. The video shows the image quality reduction (exaggerated noise) as a consequence of the sensor layer degradation caused by ion migration.

Supplementary Video 2. Evolution of the image captured by perovskite photodetector array in FPO mode. The video shows the negligible degradation of the image quality. The operational stability was gained from the suppression of ion migration effects by applying of compensation pulses of positive polarity.
